# Clinical Outcomes of Shifting from Transfemoral-First to Transradial-First Approach in Carotid Artery Stenting: A Retrospective Two-Timeframe Comparison at a Single Center

**DOI:** 10.3390/jcm13237432

**Published:** 2024-12-06

**Authors:** Taichiro Imahori, Shigeru Miyake, Ichiro Maeda, Hiroki Goto, Rikuo Nishii, Haruka Enami, Daisuke Yamamoto, Tomoaki Harada, Jun Tanaka, Junichi Sakata, Hirotoshi Hamaguchi, Nobuyuki Sakai, Takashi Sasayama, Kohkichi Hosoda

**Affiliations:** 1 Department of Neurosurgery, Kitaharima Medical Center, Ono 675-1392, Hyogo, Japan; timahori@med.kobe-u.ac.jp (T.I.); miyake@ck9.so-net.ne.jp (S.M.); i.m.996.0100@gmail.com (I.M.); hiro.ontheedge7@gmail.com (H.G.); r.nishii156@gmail.com (R.N.); rabumps@gmail.com (H.E.); dyama@med.kobe-u.ac.jp (D.Y.); harady.tomoaki@hotmail.co.jp (T.H.); jun_flatout@yahoo.co.jp (J.T.); jsakata@med.kobe-u.ac.jp (J.S.); 2 Department of Neurovascular Research, Kobe City Medical Center General Hospital, Kobe 650-0047, Hyogo, Japan; n.sakai@siren.ocn.ne.jp; 3 Department of Neurosurgery, Kobe University Graduate School of Medicine, Kobe 650-0017, Hyogo, Japan; takasasa@med.kobe-u.ac.jp; 4 Department of Neurology, Kitaharima Medical Center, Ono 675-1392, Hyogo, Japan; hirotoshi_hamaguchi@kitahari-mc.jp; 5 Department of Neurosurgery, Seijinkai Shimizu Hospital, Kyoto 615-8237, Kyoto, Japan; 6 Department of Neurosurgery, Myodani Hospital, Kobe 655-0852 Hyogo, Japan

**Keywords:** carotid artery stenosis, carotid artery stenting, transfemoral approach, transradial approach

## Abstract

**Objective:** Carotid artery stenting (CAS) has traditionally been performed using the transfemoral approach (TFA). Recently, the transradial approach (TRA) has gained attention for its lower invasiveness and reduced complication risk. This study compares outcomes between two access strategy timeframes, TFA-first and TRA-first, to evaluate how this shift influences outcomes in a real-world setting. **Methods:** A retrospective analysis of 85 CAS procedures was conducted at our institution from October 2018 to September 2024, categorizing them into TFA-first (n = 42) and TRA-first (n = 43) periods. The primary endpoint was access-related complications and 30-day perioperative events, including stroke, myocardial infarction, and mortality. The secondary endpoints included target lesion access success rate, frequency of access route conversions, procedural time, and hospital length of stay. **Results:** Baseline characteristics, including age, sex, symptomatic status, stenosis severity, plaque characteristics, and anatomical considerations, were comparable between groups. In the TFA-first period, 88% of procedures utilized TFA, and TRA was not used at all, while the remaining 12% employed the transbrachial approach (TBA). In the TRA-first period, 23% of procedures employed TFA, 60% utilized TRA, and 16% relied on TBA (*p* < 0.01). Both groups achieved a similar rate of target lesion access success (98% each) with only one conversion per group. The primary endpoint was significantly lower in the TRA-first group (0%) compared to the TFA-first group (10%, *p* = 0.04), primarily due to reduced access-site complications. Additionally, the median hospital stay was shorter in the TRA-first group at 6 days compared to 10 days (*p* = 0.02). **Conclusions:** Adopting a TRA-first strategy over TFA in CAS leads to better outcomes by improving access-site safety and reducing hospital stays. Developing TRA-specific devices could further expand the applicability of TRA in CAS.

## 1. Introduction

Carotid artery stenting (CAS) is an established endovascular intervention for carotid artery stenosis, primarily aimed at reducing the risk of ischemic stroke [1,2,3]. Traditionally, CAS has been performed via the transfemoral approach (TFA); however, TFA is associated with a significant risk of access-site complications, including bleeding and vascular injury, especially in elderly patients and those with vascular comorbidities [4,5]. These risks highlight the need for safer access alternatives. While the transbrachial approach (TBA) has been explored as an alternative to TFA, the brachial artery’s role as a major blood supply to the upper limb increases the likelihood of access-related ischemic complications, limiting its adoption as a standard approach [6,7].

The transradial approach (TRA) has gained attention as a less invasive option with a potentially lower risk of access-site complications [8]. In coronary interventions, TRA is widely preferred over TFA due to its reduced bleeding risk and vascular injury compared to TFA [9,10]. The radial artery’s smaller diameter and ease of hemostasis provide enhanced access-site safety, along with additional benefits, such as increased patient comfort, shorter hospital stays, and lower healthcare costs [10,11]. Recent studies have demonstrated similar technical success rates for CAS performed via TRA compared to TFA, with fewer access-site complications [12,13,14,15,16,17]. However, the efficacy and safety of TRA for CAS are still under investigation, and real-world clinical data remain limited.

This study aims to evaluate the impact of adopting TRA over TFA for CAS in routine clinical practice. We examined cases during periods in which TFA or TRA was the preferred access strategy at our institution, comparing access-site and perioperative complications and overall clinical outcomes. This analysis seeks to clarify the potential benefits and feasibility of using TRA as the primary approach for CAS.

## 2. Materials and Methods

### 2.1. Patient Population

**Figure 1:**
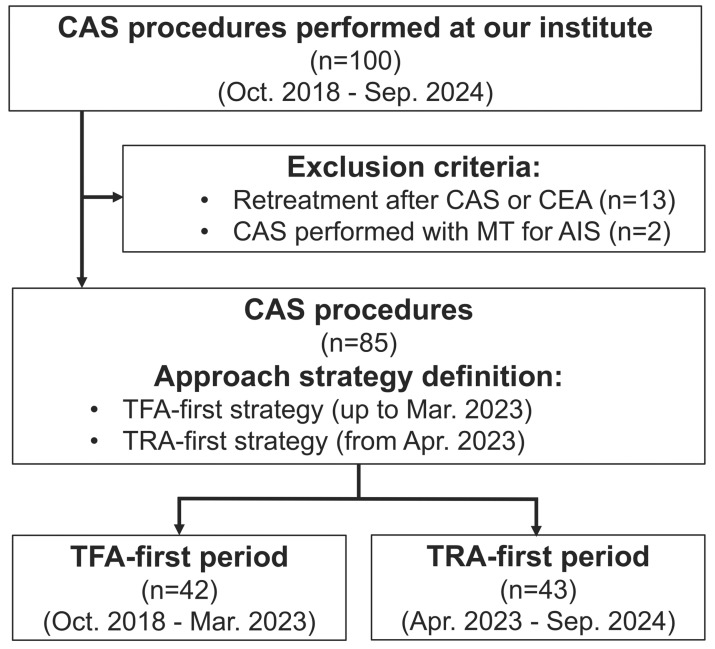
**Study flow diagram.** Flow diagram outlining patient selection criteria and approach strategies for CAS procedures performed at our institution from October 2018 to September 2024. Patients were excluded if CAS was performed as a retreatment following prior CAS or carotid endarterectomy (CEA) or if CAS was performed concurrently with mechanical thrombectomy (MT) for acute ischemic stroke (AIS). The cohort was divided into two periods based on the preferred access strategy: the TFA-first period (October 2018 to March 2023) and the TRA-first period (April 2023 to September 2024), reflecting an institutional shift from TFA to TRA as the primary strategy for CAS starting in April 2023. Abbreviations: AIS, acute ischemic stroke; CAS, carotid artery stenting; CEA, carotid endarterectomy; MT, mechanical thrombectomy; TFA, transfemoral access; TRA, transradial access.

This retrospective observational study included 100 carotid artery stenting (CAS) procedures performed at our institution between October 2018 and September 2024 (Figure 1). Cases involving retreatment after CAS or carotid endarterectomy, as well as those performed concurrently with mechanical thrombectomy for acute ischemic stroke, were excluded. After applying these criteria, 85 CAS procedures in 77 patients were included in the current analysis. Patients were categorized into two distinct periods based on the predominant approach method: the TFA-first period (October 2018 to March 2023) and the TRA-first period (April 2023 to September 2024). During the TFA-first period, 42 CAS procedures were performed in 40 patients, and during the TRA-first period, 43 procedures were performed in 39 patients. Data for this study were extracted retrospectively by reviewing patients’ medical records and imaging studies. The clinical records provided demographic information, comorbidities, symptomatic status, and treatment history, while angiographic images were reviewed to assess aortic arch anatomy, carotid stenosis severity, and procedural details. Measurements and the occurrence of perioperative events were evaluated by two experienced neurointerventionalists independently, with disagreements resolved by consensus. This study was approved by our institutional ethics committee.

### 2.2. Patient Characteristics

Baseline demographic and clinical characteristics were collected, including age, gender, comorbidities (e.g., hypertension, dyslipidemia, diabetes mellitus, coronary artery disease), symptomatic status, stenosis ratio (calculated according to NASCET criteria), and aortic arch type (classified according to Madhwals’ criteria as types 1, 2, and 3) [18,19]. Coronary artery disease was defined based on clinical diagnostic records, history of myocardial infarction, and history of or requirement for coronary artery revascularisation (e.g., percutaneous coronary intervention or coronary artery bypass grafting). Preprocedural carotid ultrasound was performed in a subset of patients to assess plaque characteristics, with a focus on features suggestive of unstable plaques: low echogenicity, plaque mobility, and the presence of ulceration [20]. Based on these baseline characteristics, perioperative risk was evaluated to determine the appropriateness of revascularisation. This evaluation informed decisions regarding procedural techniques, including the choice of access approach and distal embolic protection strategies, ensuring an individualized treatment plan tailored to each patient’s clinical and anatomical profile.

### 2.3. Antithrombotic Therapy

All patients received dual antiplatelet therapy (DAPT) with aspirin (100 mg) and clopidogrel (75 mg) prior to the procedure, with a loading dose administered when necessary. Post-procedure, patients continued DAPT for 3 to 6 months before transitioning to single antiplatelet therapy. During CAS, intravenous heparin (5000 units) was administered prior to catheter insertion, with additional doses given as needed to maintain an activated clotting time of 250–300 s.

### 2.4. CAS Procedure

The CAS procedure was performed under general anesthesia for high-risk cases and under local anesthesia for others. Access routes were selected according to the preferred strategy for each period, with adjustments based on anatomical and plaque-related factors. During the TRA-first period, a sheathless 8Fr balloon guide catheter (BGC) was commonly used, allowing proximal occlusion with a smaller puncture size. Access devices were chosen based on anatomical and plaque characteristics, resulting in the following puncture sizes: 9Fr sheath BGC (3.7 mm), 8Fr sheath BGC (3.5 mm), 8Fr sheath guide catheter (GC) (3.5 mm), 7Fr guide-sheath (GS) (3.1 mm), sheathless 8Fr BGC (2.7 mm), and 6Fr GS (2.7 mm).

The BGCs used included the 9Fr/8Fr Branchor (Asahi Intecc, Aichi, Japan), 9Fr/8Fr Cello (Medtronic, Irvine, CA, USA), and 9Fr/8Fr Optimo (Tokai Medical Products, Aichi, Japan). Guide catheters (GCs) included the 8Fr Neuro-EBU (SILUX Co., Ltd., Kawaguchi City, Japan), and guide-sheaths (GSs) included the 6Fr Axcelguide (Medikit, Tokyo, Japan) and 7Fr/6Fr Shuttle (Cook, Minneapolis, MN, USA). Distal protection was achieved using filter or balloon devices, with proximal protection as needed. Protection devices included FilterWire EZ (Stryker, Fremont, CA, USA), SpiderFX (Medtronic), Carotid GuardWire PS (Medtronic), and Optimal Wire (Tokai Medical Products), along with the listed BGCs.

Stents, such as Casper (MicroVention, Tustin, CA, USA), Precise (Cordis, Miami Lakes, FL, USA), and Carotid Wallstent (Stryker), were chosen based on lesion characteristics. Procedure time was defined as the duration from initial puncture to sheath removal. Closure devices for TFA procedures included Angio-Seal (St. Jude Medical, St. Paul, MN, USA) and Perclose Proglide (Abbott Vascular, Lake Bluff, IL, USA). Hemostasis for TBA and TRA procedures was achieved with compression bands such as PreludeSYNC (Merit Medical Systems, South Jordan, UT, USA) and Bleed Safe (Medikit).

### 2.5. Outcome Measurement

The primary endpoint was a composite of access-related complications and 30-day periprocedural adverse events. Access-related complications included severe bleeding, defined as a hemoglobin decrease of >2 g/dL or requiring transfusion, pseudoaneurysm formation, and symptomatic arterial occlusion. Periprocedural adverse events included cerebrovascular events, myocardial infarction, and death. Stroke was defined as focal neurological symptoms persisting for over 24 h and was further classified into major stroke (NIHSS ≥ 9) and minor stroke (NIHSS < 9). Secondary endpoints included the target lesion access success rate, frequency of access route conversions, procedure time, and hospital length of stay.

### 2.6. Statistical Analyses

Continuous variables were presented as medians with interquartile ranges, while categorical variables were expressed as percentages and counts. Welch’s *t*-test was used to compare continuous variables, and the chi-squared test was used to compare categorical variables. Statistical significance was set at *p* < 0.05. All analyses were conducted using JASP (Version 0.19.0).

## 3. Results

### 3.1. Patient Characteristics

Baseline characteristics, including age, gender, comorbidities, symptomatic lesion status, stenosis ratio, and anatomical features, were similar between the TFA-first period group (n = 42) and TRA-first period group (n = 43), with no significant differences observed (Table 1). Specifically, lesion laterality, aortic arch type distribution, and the presence of a bovine arch were comparable between groups, indicating a balanced cohort that facilitates comparability of outcomes between access approach strategies. Preprocedural carotid ultrasound findings were available for most patients, except for those requiring urgent intervention, and no significant differences in plaque characteristics were observed between groups.

### 3.2. Procedural Details

**Figure 2:**
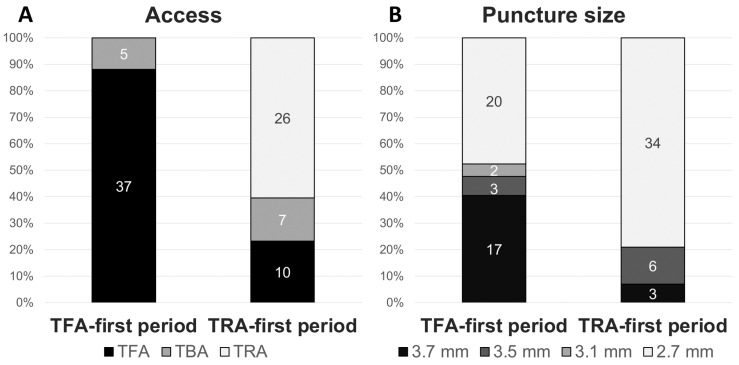
**Distribution of access sites and puncture sizes across TFA-first and TRA-first periods.** Bar charts illustrating the use of different vascular access sites and puncture sizes across the TFA-first and TRA-first periods. The (**A**) displays the percentage distribution of TFA, TBA, and TRA for each period, while the (**B**) shows the distribution of various puncture sizes (3.7 mm, 3.5 mm, 3.1 mm, and 2.7 mm) across both periods. Abbreviations: TBA, transbrachial access; TFA, transfemoral access; TRA, transradial access.

The distribution of access approaches that were actually applied differed significantly between the two periods (Figure 2A, Table 2). In the TFA-first period, TFA was used in 88% of cases, TBA in 12%, and TRA was not used. In contrast, during the TRA-first period, TRA was used in 60% of cases, TFA in 23%, and TBA in 16% (*p* < 0.01). The reasons for selecting TFA or TBA during the TRA-first period included factors such as a narrow radial artery, anatomical conditions, and plaque characteristics.

The success rate for accessing the target lesion was high across both periods, achieving 98% with only one conversion required in each group. In the TFA-first period, one case required conversion from TBA to TFA due to difficulty in catheter navigation. In the TRA-first period, one case was converted from TRA to TBA due to severe pain caused by radial artery spasms during guide catheter manipulation. The choice of anesthesia remained consistent across periods, with local anesthesia used in 88% of cases in the TFA-first period and 93% in the TRA-first period (*p* = 0.44). In cases using TRA, patients more frequently reported pain, and almost all of them received analgesics.

**Figure 3:**
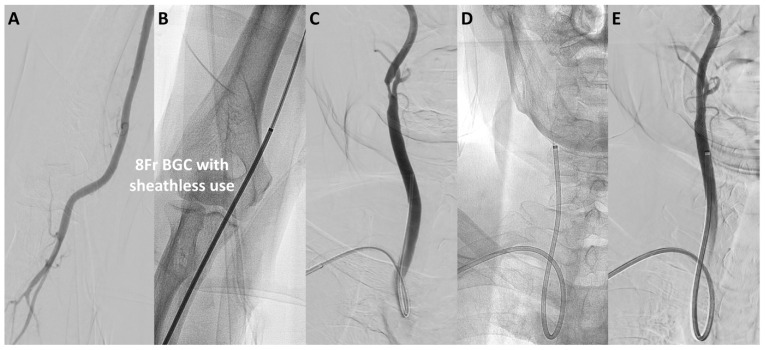
**Representative case of TRA CAS using a sheathless 8Fr BGC in favorable approach anatomy.** A representative case of CAS performed via the radial artery using a sheathless 8Fr BGC in favorable approach anatomy. (**A**,**B**) show the insertion of the 8Fr BGC (outer diameter 2.7 mm) into the radial artery using a sheathless approach (puncture size 2.7 mm). (**C**,**D**) demonstrate smooth navigation of the BGC into the right common carotid artery (CCA). (**E**) shows successful stent placement. Abbreviations: CAS, carotid artery stenting; CCA, common carotid artery; BGC, balloon guide catheter; TBA, transbrachial access; TRA, transradial access.

**Figure 4:**
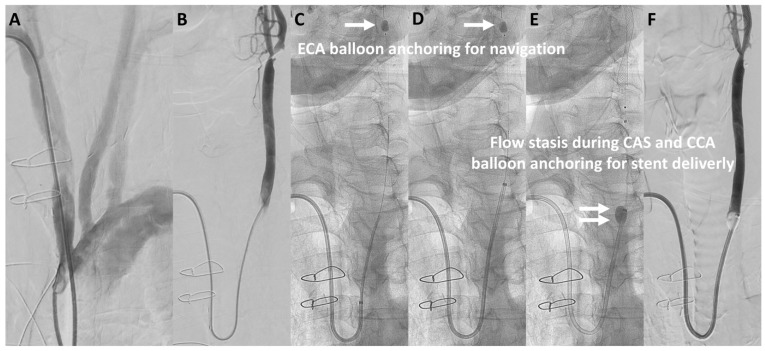
**Representative case of TRA CAS in challenging approach anatomy.** A representative case of TRA CAS using a sheathless 8Fr BGC in challenging approach anatomy. Aortography shows a steep angle between the brachiocephalic artery and the left common carotid artery (CCA), suggesting potential difficulty in guiding a catheter to the left CCA using TRA or TBA (**A**). Due to the presence of unstable plaque, a sheathless 8Fr BGC was chosen to reduce distal embolization risk. (**B**) illustrates the selection of the left CCA using a Simmons-type inner catheter. In (**C**,**D**), a distal protection balloon is inflated in the external carotid artery (ECA) to serve as an anchor, facilitating navigation of the guiding catheter over the sharp curve to the target site (arrow: the ECA balloon). In (**E**,**F**), during CAS, the distal protection balloon is inflated in the internal carotid artery (ICA), and the 8Fr BGC balloon is inflated to achieve flow stasis and stabilize stent delivery (double arrows: the 8Fr BGC balloon). Abbreviations: CAS, carotid artery stenting; CCA, common carotid artery; ECA, external carotid artery; ICA, internal carotid artery; BGC, balloon guide catheter; TRA, transradial access.

The median puncture size was reduced from 3.1 mm in the TFA-first period to 2.7 mm in the TRA-first period (*p* < 0.01; Figure 2B). Specifically, in the TFA-first period, the most commonly used devices were the 9Fr sheath BGC (40%) and the 6Fr guide sheath (GS) (48%). In the TRA-first period, however, the sheathless 8Fr BGC was widely used, accounting for 70% of cases, with minimal use of larger devices (*p* < 0.01). The widespread adoption of the sheathless 8Fr BGC during the TRA-first period was primarily due to its advantages in managing unstable plaque and providing enhanced support during device navigation. Representative cases of TRA CAS using the sheathless 8Fr BGC are illustrated in Figure 3 and Figure 4. Figure 3 shows a favorable anatomy case with smooth BGC navigation, while Figure 4 demonstrates a challenging anatomy case where BGC use was desired due to unstable plaque; however, balloon anchoring in the external carotid artery (ECA) was required to guide the BGC into position.

While it is important to consider the differences in available devices across different periods, the use of distal protection devices also varied between periods. In the TRA-first period, filter-type distal protection devices were more frequently used (79% vs. 64%), while balloon-type protection was less common (14% vs. 36%) (*p* = 0.02). Proximal protection alone was used in 7% of TRA-first cases, whereas it was not employed in the TFA-first period.

Stent selection also differed between periods. The Wallstent was the most frequently used stent in both periods, with increased use in the TRA-first period (88% vs. 69%). The Precise stent was used more often in the TFA-first period (31% vs. 9%), and the Casper stent was used only in one TRA-first case (*p* = 0.03).

Finally, the procedural time was significantly shorter in the TRA-first period, with a median of 81 min compared to 121 min in the TFA-first period (*p* < 0.01, Table 2).

### 3.3. Procedural Outcomes

**Figure 5:**
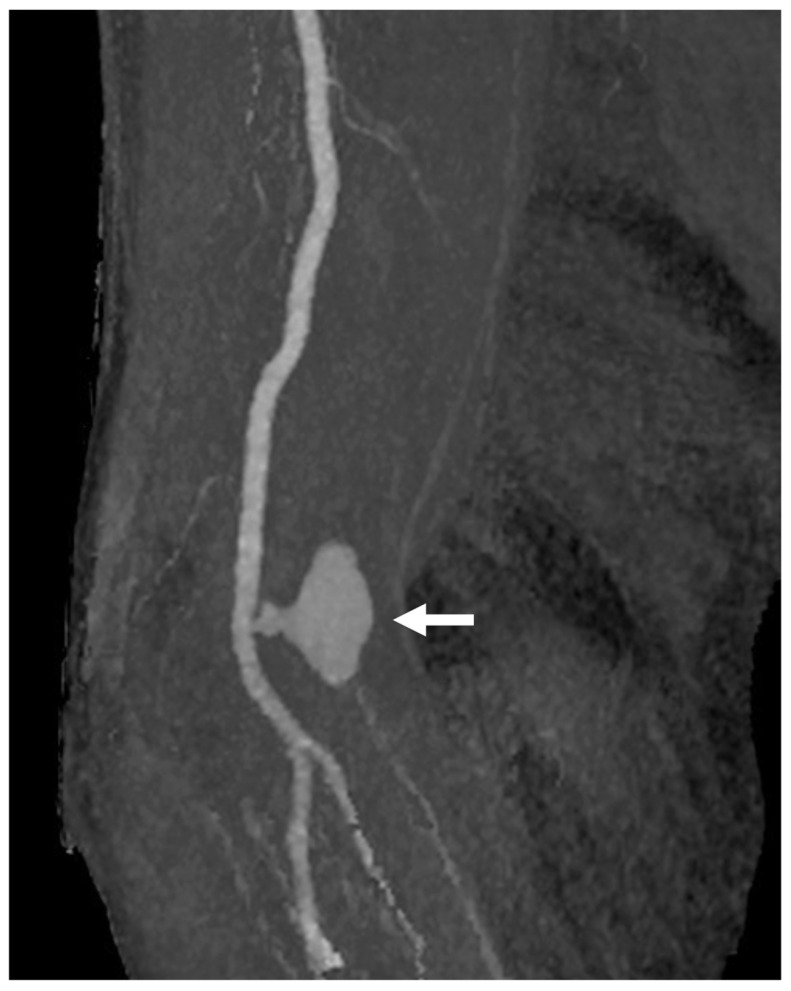
**Pseudoaneurysm formation following TBA CAS.** CTA demonstrates pseudoaneurysm formation as a complication following TBA CAS. In this case, a 6Fr guide sheath (outer diameter 2.7 mm) was inserted into the brachial artery (puncture size 2.7 mm). The pseudoaneurysm was subsequently treated with a thrombin injection (arrow: the psheudoaneurysm). Abbreviations: CAS, carotid artery stenting; CTA, computed tomography angiography; TBA, transbrachial access.

**Figure 6:**
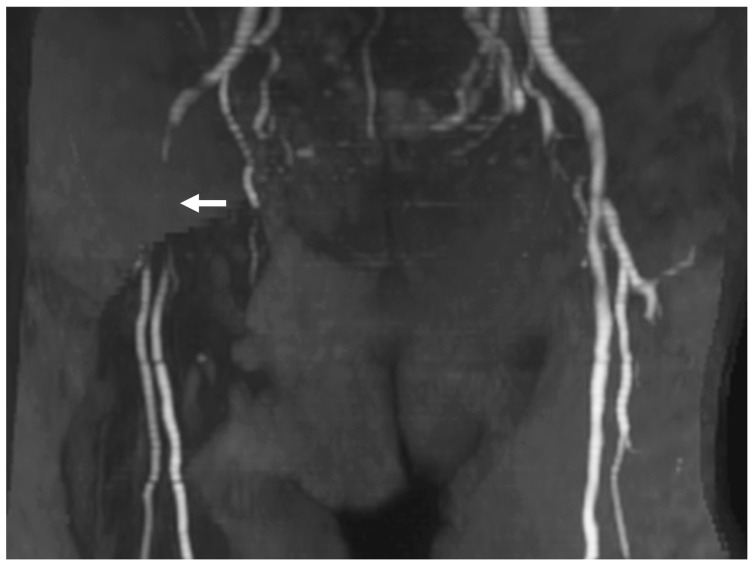
**Femoral artery occlusion following TFA CAS.** MRA demonstrates femoral artery occlusion after TFA CAS. In this case, a 9Fr BGC (outer diameter 3 mm) was introduced through a 9Fr sheath (outer diameter 3.7 mm) placed in the femoral artery (puncture size 3.7 mm). The occlusion caused symptoms of coldness, pain, and cyanosis (arrow: the femoral artery occlusion). Although conservative treatment led to some improvement, symptoms persisted. Abbreviations: CAS, carotid artery stenting; BGC, balloon guide catheter; MRA, magnetic resonance angiography; TFA, transfemoral access.

The success rate for CAS was 100% in both periods (Table 3). However, the primary endpoint, a composite measure of access-related complications and 30-day periprocedural adverse events, was significantly lower in the TRA-first period (0%) compared to the TFA-first period (10%, *p* = 0.04). Access-related complications in the TFA-first period included severe hematoma, pseudoaneurysm formation, and symptomatic arterial occlusion, none of which occurred in the TRA-first period (Figure 5 and Figure 6). Additionally, a post-CAS complication of ischemic stroke occurred in one case during the TFA-first period, while no such events were observed in the TRA-first period.

The median hospital length of stay was also significantly shorter in the TRA-first period, with a median of 6 days compared to 10 days in the TFA-first period (*p* = 0.02, Table 3).

## 4. Discussion

This study evaluated the clinical impact of shifting from the TFA-first to the TRA-first strategy in CAS within a real-world setting. Rather than a direct comparison of TFA and TRA, this study provides a comprehensive evaluation of access strategies during two distinct periods, reflecting the practical challenges of implementing TRA as the primary approach. The findings indicate that adopting a TRA-first strategy was associated with significantly reduced access-site complications and shorter hospital stays compared to the preceding TFA-first period while maintaining clinical efficacy. These results underscore the potential benefits of TRA as a preferred strategy in suitable cases but also highlight the technical and anatomical challenges that currently limit its universal application. Further advancements in TRA-specific devices and training may help expand its feasibility and adoption in CAS.

### 4.1. Effectiveness and Safety of TRA

In both the TRA-first and TFA-first periods, the success rate for CAS procedures was high, demonstrating the effectiveness of CAS regardless of the access approach. However, in terms of safety, the incidence of complications in the TRA-first period was 0%, significantly lower than the 10% observed in the TFA-first period (*p* = 0.04), largely due to access-related complications associated with TFA and TBA. This reduction is consistent with previous studies, suggesting that TRA has the potential to reduce risks associated with access sites [14,15,16,17]. Interestingly, access-related complications were relatively low during the TFA-first period, likely because sheathless guiding catheters were used in approximately half of the cases, which may have helped reduce puncture size and contributed to a lower complication rate.

### 4.2. Effect on Hospital Stay Reduction

The TRA-first strategy contributed to a shorter hospital stay, with a median of 6 days compared to 10 days in the TFA-first period (*p* = 0.02). This reduction in hospital stay likely reflects the decreased risk of access-related complications, shorter procedural time, and the possibility of early mobilization following TRA. Consistent with previous findings from coronary interventions, a shorter hospital stay facilitates faster social reintegration and reduces healthcare costs, reinforcing the clinical value of adopting a TRA-first strategy for CAS [11].

### 4.3. Feasibility of TRA Applications and Challenges

While TRA offers several advantages, it is not universally applicable, as anatomical constraints may limit its use. In this study, TRA was applied in 60% of cases during the TRA-first period; however, for the remaining cases, TFA or TBA approaches were selected primarily due to anatomical factors. Consistent with previous studies, cases on the left side involving complex aortic arch anatomy presented higher procedural challenges [21,22]. Expanding the applicability of TRA will require enhanced operator training and the adoption of standardized techniques. Additionally, new devices and technologies, such as Simmons-type guiding catheters, which allow stable access even in complex anatomical conditions, are essential for the broader adoption of TRA [23,24,25].

### 4.4. Sheathless BGC Use in TRA

One of the key factors contributing to the favorable outcomes of TRA in this study was the extensive use of sheathless 8Fr BGCs. In TRA cases, the primary device was the sheathless BGC, which facilitated a reduced puncture size and may have contributed to improved clinical outcomes. Although prior reports have commonly utilized non-balloon guiding sheaths for TRA, recent studies increasingly emphasize the advantages of sheathless BGCs. Sheathless BGCs offer benefits such as reliable flow stasis and enhanced device stability [26,27,28]. These features improve procedural efficiency and help reduce complications. However, to fully address the anatomical limitations specific to TRA, further optimization in TRA-dedicated devices is essential.

### 4.5. Study Limitations and Future Directions

This study has several limitations. Firstly, as a retrospective observational study conducted at a single center, the generalizability of the findings may be restricted. Additionally, the sample size is relatively small, necessitating further large-scale studies to validate our findings. Another limitation is the inclusion of multiple access approaches (TFA, TRA, and TBA) within the analysis. While this reflects real-world clinical practice, it introduces variability that may complicate the direct comparison between TFA and TRA. However, we believe that this approach highlights the current challenges in achieving universal TRA applicability and underscores the need for further advancements in TRA-specific devices and operator training. Furthermore, while data evaluation was performed independently by two experienced neurointerventionalists, a fully blinded assessment was not feasible due to the retrospective nature of the study. The accessibility of procedural details in medical records and imaging data may introduce potential bias in the evaluation of perioperative outcomes. In the future, randomized controlled trials focusing exclusively on TFA and TRA may provide additional clarity regarding their comparative efficacy and safety.

## 5. Conclusions

This study supports adopting a TRA-first strategy for CAS, showing significant reductions in access-site complications and hospital stays without compromising efficacy. TRA provides a safer alternative to TFA; however, anatomical limitations suggest a need for further advancements in TRA-specific devices. These findings advocate for the broader use of TRA in CAS to improve patient safety and procedural outcomes.

## Figures and Tables

**Table 1:** Patient characteristics.

	TFA-First Period (n = 42)	TRA-First Period (n = 43)	*p*-Value
Age, years	76 (71–81)	75 (72–84)	0.73
Gender, male	86% (36)	93% (40)	0.27
Cormobidities			
Hypertension	76% (32)	71% (30)	0.67
Dyslipidemia	62% (26)	53% (23)	0.43
Diabetes mellitus	33% (14)	47% (20)	0.22
Coronary artery disease	26% (11)	23% (10)	0.75
Symptomatic lesion	43% (18)	44% (19)	0.90
Stenosis ratio	78 (74–82)	78 (71–82)	0.39
Carotid ultrasound †			
Low echogenicity	34% (14/41)	25% (9/36)	0.38
Mobility	10% (4/41)	8% (3/36)	0.83
Ulceration	12% (5/41)	14% (5/36)	0.83
Target side			0.91
Right	55% (23)	53% (23)	
Left	45% (19)	47% (20)	
Aortic arch type			0.52
Type 1	29% (12)	21% (9)	
Type 2	29% (12)	40% (17)	
Type 3	43% (18)	40% (17)	
Bovine arch	10% (4)	14% (6)	0.53

Continuous variables are presented as median (interquartile range), and categorical variables are presented as % (number). *p*-values were calculated using Welch’s *t*-test for continuous variables and the chi-squared test for categorical variables. Statistical significance was set at *p* < 0.05 † Preprocedural carotid ultrasound was available for 41/42 TFA-first and 36/43 TRA-first procedures, as urgent cases did not allow time for ultrasound. Abbreviations: TFA, transfemoral approach; TRA, transradial approach; n, number of patients.

**Table 2:** Procedural details.

	TFA-First Period (n = 42)	TRA-First Period (n = 43)	*p*-Value
Anesthesia, local	88% (37)	93% (40)	0.44
Access			<0.01 *
TFA	88% (37)	23% (10)	
TBA	12% (5)	16% (7)	
TRA	0% (0)	60% (26)	
Success of access to the target lesion			0.37
Success	98% (41)	98% (42)	
Conversion to TFA	2% (1)	0% (0)	
Conversion to TBA	0% (0)	2% (1)	
Sheath/guiding catheter (puncutre size, mm)			<0.01 *
9Fr sheath BGC (3.7)	40% (17)	7% (3)	
8Fr sheath BGC (3.5)	2% (1)	14% (6)	
8Fr sheath GC (3.5)	5% (2)	0% (0)	
7Fr GS (3.1)	5% (2)	0% (0)	
Sheathless 8Fr BGC (2.7)	0% (0)	70% (30)	
6Fr GS (2.7)	48% (20)	9% (4)	
Puncutre size	3.1 (2.7–3.7)	2.7 (2.7–2.7)	<0.01 *
Distal protection			0.02 *
Filter	64% (27)	79% (34)	
Balloon	36% (15)	14% (6)	
Proximal protection only	0% (0)	7% (3)	
Stent			0.03 *
Casper	0% (0)	2% (1)	
Precise	31% (13)	9% (4)	
Wallstent	69% (28)	88% (38)	
Procedural time, min	121 (104–142)	81 (57–95)	<0.01 *

Continuous variables are presented as median (interquartile range), and categorical variables are presented as % (number). *p*-values were calculated using Welch’s *t*-test for continuous variables and the chi-squared test for categorical variables. Statistical significance was set at *p* < 0.05. * *p* < 0.05, indicates statistical significance. Abbreviations: TFA, transfemoral approach; TBA, transbrachial approach; TRA, transradial approach; BGC, balloon-guiding catheter; GC, guiding catheter; GS, guide-sheath; n, number of patients.

**Table 3:** Procedural outcomes.

	TFA-First Period (n = 42)	TRA-First Period (n = 43)	*p*-Value
Success of CAS procedure	100% (42)	100% (43)	1.00
Primary endpoint (the composite of access-related and post-CAS procedure complication)	10% (4)	0% (0)	0.04 *
Access-related complication			
The following composite	7% (3)	0% (0)	0.07
Severe hematoma	2% (1)	0% (0)	0.30
Pseudoaneurysm formation	2% (1)	0% (0)	0.30
Symptomatic artery occlusion	2% (1)	0% (0)	0.30
Post-CAS procedure complication			
The following composite	2% (1)	0% (0)	0.31
Major cerebrovascular events	2% (1)	0% (0)	0.31
Minor cerebrovascular events	0% (0)	0% (0)	1.00
Myocardial infarction	0% (0)	0% (0)	1.00
Mortality	0% (0)	0% (0)	1.00
Hospital stay, days	10 (9–12)	6 (4–12)	0.02 *

Continuous variables are presented as median (interquartile range), and categorical variables are presented as % (number). *p*-values were calculated using Welch’s *t*-test for continuous variables and the chi-squared test for categorical variables. Statistical significance was set at *p* < 0.05. * *p* < 0.05, indicates statistical significance. Abbreviations: CAS, carotid artery stenting; TFA, transfemoral approach; TRA, transradial approach; n, number of patients.

## Data Availability

The data presented in this study are available on request from the corresponding author.
